# Genetic diversity of an Azorean endemic and endangered plant species inferred from inter-simple sequence repeat markers

**DOI:** 10.1093/aobpla/plu034

**Published:** 2014-06-26

**Authors:** Maria S. Lopes, Duarte Mendonça, Sílvia X. Bettencourt, Ana R. Borba, Catarina Melo, Cláudio Baptista, Artur da Câmara Machado

**Affiliations:** Biotechnology Centre of Azores, Associated Laboratory Institute for Biotechnology and Bioengineering, University of Azores, Rua Capitão João D'Ávila, 9700-042 Angra do Heroísmo, Portugal

**Keywords:** Azores, endemism conservation, germplasm management, molecular marker, phylogeography, population genetics.

## Abstract

Knowledge of the levels and distribution of genetic diversity is important for designing conservation strategies for threatened and endangered species so as to guarantee sustainable survival of populations and to preserve their evolutionary potential. *Picconia azorica* is a valuable Azorean endemic species recently classified as endangered. To contribute with information useful for the establishment of conservation programmes, the genetic variability and differentiation among 230 samples from 11 populations collected in three Azorean islands was accessed with eight inter-simple sequence repeat markers. A total of 64 polymorphic loci were detected. The majority of genetic variability was found within populations and no genetic structure was detected between populations and between islands. Also the coefficient of genetic differentiation and the level of gene flow indicate that geographical distances do not act as barriers for gene flow. In order to ensure the survival of populations *in situ* and *ex situ* management practices should be considered, including artificial propagation through the use of plant tissue culture techniques, not only for the restoration of habitat but also for the sustainable use of its valuable wood.

## Abstract (precis)

*Picconia azorica* is an endangered endemic species of the Azores whose hard and high density wood is very appreciated for the production of toys, agricultural tools, furniture and religious statuary. Its renewed economic interest represents a good opportunity for establishing conservation programmes. To contribute with information useful for the decision making we performed the genetic analysis of 230 samples from 11 populations collected in three Azorean islands. The majority of the genetic variability was found within populations and no genetic structure was detected between populations and between islands, indicating that the oceanic barriers do not greatly affect gene flow.

## Introduction

Knowledge of genetic variability, together with information about demography, reproductive biology and dynamics, is very important when establishing any conservation and management programme (Newton *et al.* 1999; Francisco-Ortega *et al.* 2000; Juan *et al.* 2000; Frankham 2003; Jamieson 2007; Silva *et al.* 2011) that aims to preserve genetic variability within and among populations and consequently safeguard their potential for adaptation (Eriksson 2001; Silva *et al.* 2011). Information about genetic diversity patterns may also give insight into the evolutionary history of a taxon, providing the means to assess the future risk of diversity erosion (Neel and Ellstrand 2003).

The development of molecular genetic methods has been a significant advance providing tools for answering questions on the diversity among flora and fauna and to help define strategies for conservation purposes. Inter-simple sequence repeat (ISSR) markers offer the possibility of randomly scanning the whole genome (Mariette *et al.* 2001) as no sequence information or prior genetic studies are required (Zietkiewicz *et al.* 1994), and have proved to be valuable, efficient and cost-effective tools in the characterization and evaluation of genetic diversity within and between species and populations of several endemic and endangered plant species (Palacios and González-Candelas 1997; Delgado *et al.* 1999; Rossetto *et al.* 1999; Li and Ge 2001; Lee *et al.* 2003; Torres *et al.* 2003; Bahulikar *et al.* 2004; Chen *et al.* 2004; Segarra-Moragues *et al.* 2005; Guasmi *et al.* 2012). Inter-simple sequence repeats are DNA sequences delimited by two inverted SSR and are especially useful in detecting diversity in closely related, or even clonal, individuals (Zietkiewicz *et al.* 1994). The use of just a single PCR primer to amplify ISSR sequences between simple sequence repeat (SSR) provides multilocus patterns that are very reproducible, abundant and polymorphic in plant genomes (Zietkiewicz *et al.* 1994; Bornet and Branchard 2001; Bornet *et al.* 2002, 2004), making this technology a good reliable molecular tool to determine the genetic diversity of Azorean endemic and endangered species.

The archipelago of the Azores is located in the North Atlantic Ocean between latitudes 36°55′N and 39°43′N, and longitudes 24°46′W and 31°16′W. It is composed of nine volcanic islands divided into three groups: the eastern (São Miguel and Santa Maria), the central (Terceira, Graciosa, São Jorge, Pico and Faial) and the western group (Flores and Corvo). From the about 200 species native to the Azorean flora only 70 have been described as endemic for the Azores (Schaefer 2003, 2005), representing 7.2 % of the Azorean flora (Borges *et al.* 2005). The abusive use of Azorean forests for over five centuries, the expansion of pasture and agricultural fields and the introduction of exotic/imported species led to a substantial reduction of the natural native forest populations. Only recently there has been an increased interest in the endemic forest species for reforestation, particularly of rare species and species with a high wood quality (Ferreira and Eriksson 2006).

*Picconia azorica* (Oleaceae Family), locally named *pau-branco*, is a xerophytic, evergreen shrub or a small tree, endemic to the Azores archipelago. This species grows up to 8 m tall and has simple, lanceolate to ovate, opposite leaves with entire margins; it flowers from March to July, producing small white flowers in axillary clusters and its fleshy fruits are dark blue drupes (Martins *et al.* 2011). Although *P. azorica* has been present in all nine Azorean islands (Frutuoso 1583), currently is scattered in small patches of coastal forests and marginal sites (Schaefer 2003). Clearly, the over-exploitation of its appreciated wood for manufacturing toys, agricultural tools, furniture and religious statuary, as well as human disturbance that promoted habitat degradation, expansion of agricultural land, deforestation and introduction of aggressive exotic species led this species to becoming almost extinct in some islands (Martín *et al.* 2008). In fact, it is extinct in Graciosa island and near to extinction in São Miguel and Terceira islands (Cardoso *et al.* 2008; Silva *et al.* 2010; Ferreira *et al.* 2011), thus being a priority Azorean endemic species for conservation, listed as endangered (EN B1 + 2c) on the IUCN Red List (2013) and protected according to the Directive Habitats (Annexes II and IV) (European Commission 1992) and the Bern Convention (Annex I) (Council of Europe 1993). *Picconia azorica* is one of the two residual species of the *Picconia* genera which became extinct in continental land. However the Azores, together with Madeira and the Canary islands, are considered refuge areas for this genus. Therefore, *P. azorica* due to its fragile status demands urgent specific management and conservation measures for restoration of depleted natural populations (Martín *et al.* 2008). Ecological restoration plans to prevent the erosion of *P. azorica* genetic resources are necessary, primarily because of its endangered status, for its biological and economical relevance and for its major ecological importance. Unfortunately, there are limited numbers of studies on the Azorean endemic forests and no detailed characterization about the biology and management of *P. azorica* has been made so far (Ferreira *et al.* 2011). For the genetic characterization of *P. azorica,* chloroplast markers were used under phylogenetic and phylogeographical perspectives, due to their uniparental inheritance, revealing absence of geographical structure and limited intra-specific genetic diversity (Ferreira *et al.* 2011), suggesting the need for a sound assessment of the species genetic structure at the nuclear level. Only recently a limited number of SSRs were isolated from *P. azorica* and used for the characterization of different populations (Martins *et al.* 2013), showing high levels of intra-population diversity and low genetic differentiation between populations. Simple sequence repeats are codominant markers that reveal a high number of alleles, still the variation detected is pre-determined at the sequence sites and the number of analysed loci in diversity studies is usually low (Mariette *et al.* 2001). In our study we access the genetic variability and differentiation, as well as phylogeographical patterns within different populations of São Miguel, Terceira and Pico islands of such an endangered endemic Azorean species, by using ISSR markers, as conservation should be based on genetic diversity at the whole-genome level (Mariette *et al.* 2001). These islands were selected as they are the most populated ones and therefore more susceptible to human habitat disturbance and also because Pico is the island with more area forested with *P. azorica* and since the earliest colonization of the archipelago an exporter of this valuable wood (Frutuoso 1583).

## Methods

*Picconia azorica* leaf samples were collected from 11 naturally occurring populations from three Azorean Islands: São Miguel, Terceira and Pico (Table 1). Populations Caloura (CL), Lombo Gordo (LG), Ribeira Quente (RQ) and Tronqueira (TR) were collected in São Miguel Island located in the eastern group of the archipelago. The remaining populations were collected from islands located in the central group: Pico [Alto de São Roque (ASR), Cais do Mourato—Santana (CM), Candelária (CD), Piedade (PD) and Santo Amaro (SA)] and Terceira [Pico do Boi (PB) and Serreta (SER)] **[see Supporting Information]**. Geographical coordinates of all stations of origin were recorded using a hand-help GPS navigator. Samples were collected randomly across the distribution area with at least a minimal distance of 20 m. Efforts were made that the number of individuals collected was correlated to the dimension of each population. However, some populations were difficult to access (TR) or were very small (CL), in which case sampling was limited. Leaf samples were weighed and stored at −80 °C.

**Table 1.** Populations of *P. azorica* from S. Miguel, Pico and Terceira islands used in this study. *N*, number of individuals sampled. For population locations, see **Supporting Information**.

Island	Population	Code	*N*	Altitude range (m)
S. Miguel	Caloura	CL	4	30–50
	Lombo Gordo	LG	16	20–270
	Ribeira Quente	RQ	37	30–100
	Tronqueira	TR	7	560–615
Pico	Alto de São Roque	ASR	14	240–430
	Cais do Mourato – Santana	CM	37	15–115
	Candelária	CD	17	30–220
	Piedade	PD	27	10–320
	Santo Amaro	SA	24	10–50
Terceira	Pico do Boi	PB	19	645–690
	Serreta	SER	28	140–230

DNA was extracted by downscaling the protocol described by Fabbri *et al.* (1995) using 100 mg of leaves, so that extraction could be performed with 800 μL of CTAB extraction buffer in 2 mL microtubes.

A total of 19 anchored ISSR primers from the University of British Columbia (UBC, Vancouver, Canada) were firstly tested in a batch of 48 samples. From these, 8 that produced clear, polymorphic, reproducible bands across two repetitions of each ISSR assay were selected (Table 2) and used for genotyping all the 230 samples. Additionally three samples of *Picconia excelsa*, from Madeira island, were also genotyped to be used as an outgroup for the phylogenetic analysis of individuals.

**Table 2.** Primers used for ISSR amplification of *P. azorica*.^a^ Melting temperature.

ISSR			Bands		
Primer name	Primer sequence	*T*_m_ (°C)^a^	Readable	Polymorphic	% Polymorphic
UBC807	(AG)_8_T	52	15	12	80.00
UBC808	(AG)_8_C	54	12	10	83.33
UBC811	(GA)_8_C	54	9	7	77.77
UBC826	(AC)_8_C	54	6	3	50.00
UBC834	(AG)_8_YT	54	8	6	75.00
UBC836	(AG)_8_YA	56	4	2	50.00
UBC840	(GA)_8_YT	54	10	10	100.00
UBC842	(GA)_8_YG	56	15	14	93.33
		Average	10	8	76.18
		Total	79	64	81.01

Polymerase chain reactions were carried out in a total volume of 25 μL containing 20 ng DNA, 0.2 μM of primer, 0.2 mM of each dNTPs and 1 U of DreamTaq DNA polymerase (Fermentas) in reaction buffer. Amplification was performed in a UNO II Biometra thermocycler with 5 min denaturation at 94 °C, followed by 35 cycles of 30 s denaturation at 96 °C, 45 s annealing at the appropriate melting temperature (Table 2), 1 min 30 s elongation at 72 °C and a final 20 min elongation step at 72 °C. Amplification products were separated for 3 h at 120 V on a 2 % TAE agarose gel stained with SybrGreen Premium (NZYTech) along with GeneRuler 1 kb Plus DNA ladder (Fermentas) for estimation of the molecular size of the amplified fragments. Gels were recorded under ultraviolet light with a GelDoc XR+ system and analysed with the Image Lab 3.0 software (BioRad). To confirm that fragments were being consistently amplified, one sample was replicated across all runs. Furthermore, to minimize the effects of electrophoresis and staining on band variability two replicate experiments were carried out for each ISSR primer–sample combination. In every case the banding patterns on the first and second amplification/gels were identical. To ensure that neither self-amplification nor contamination was occurring, negative controls were also prepared.

Amplified bands of size ranging from 0.4 to 2 kb were scored manually as present (1) or absent (0) and compiled into a data matrix. As we were studying an Azorean endemic species monomorphic loci were considered for the analysis of genetic parameters as suggested by Ayres and Ryan (1999) and Ellstrand and Elam (1993).

Genetic diversity was measured at individual, population and island levels. At the individual level, a cladogram of all the 233 samples based on the similarity analysis of fingerprint patterns was drawn using the unweighted pair group method arithmetic average (UPGMA) algorithm using Dice's coefficient as implemented in NTSYSpc (Rohlf 1992) and was plotted into a dendrogram.

To learn about the genetic diversity among populations and among islands, we calculated Nei's coefficient (*h*) (Nei 1972), Shannon's information index (*I*) (Shannon and Weaver 1949), unbiased Nei's genetic distance (Nei 1978), coefficient of genetic differentiation between populations (*G*_ST_) (Nei 1973) and gene flow (*N_m_*) (Slatkin and Barton 1989) in the software POPGENE 1.31 (Yeh *et al.* 1999) with 1000 permutations. Different analysis indexes were used to enhance the significance of the results. The estimates of genetic diversity obtained by Shannon's index (*I*) and Nei's gene diversity (*h*) were compared by Pearson's rank coefficient correlation (Sokal and Rohlf 1995) using IBM SPSS statistics 20 software and to compare analysis between marker systems, as these should not be based on the comparison of levels of diversity within populations, but on the comparison of the ranking of different populations by correlation analysis (Mariette *et al.* 2001).

The number of different alleles (*N*_a_) and the number of effective alleles (*N*_e_) (Brown and Weir 1983) were also computed with POPGENE 1.31 (Yeh *et al.* 1999) to allow comparison of populations with different sample sizes and where the number and distribution of alleles differ (Ojango *et al.* 2011).

To learn about the partitioning of genetic variabilty among islands, among populations and among individuals (Excoffier *et al.* 1992; Huff *et al.* 1993), an analysis of molecular variance (AMOVA) was performed with GenAlEx 6.5 (Peakall and Smouse 2012) by resampling 999 times.

Genetic differentiation among populations (*φ*_PT_) based on the Euclidean distances (Huff *et al.* 1993) and among individuals was calculated with GenAlEx 6.5. The former were plotted in a tree using the UPGMA method from the NEIGHBOR module from Phylip 3.695 (Felsenstein 2005) and the latter were used to carry out a principal coordinate analysis (PCoA) in GenAlEx 6.5. The Mantel test computed with GenAlEx 6.5 with 999 replications was used to evaluate correlations between the pairwise Euclidean genetic distance of individuals and their geographical distance, and between pairwise genetic differentiation (*φ*_PT_) and geographical distances of each population (Huff *et al.* 1993). This software was also used to estimate the number of migrants (Frankham *et al.* 2002).

To investigate the genetic structure and the degree of admixture between each sample and between the 11 Azorean populations, the Bayesian clustering procedure of STRUCTURE (Pritchard *et al.* 2000) was used by running an admixture model with correlated frequencies between populations. A 10 000 initial burn-in was used, followed by 10 000 MCMC iterations as suggested by Evanno *et al.* (2005) with 10 independent replicates each. The tested number of clusters varied from 1 to 14 (the number of populations plus three). The most likely number of clusters (*K*) was estimated by using the maximum value of *L*(*K*) and by calculating Δ*K* (Evanno *et al.* 2005). A mean of the 10 permuted matrices was estimated using CLUMPP (Jakobsson and Rosenberg 2007) and the *LargeKGreedy* algorithm. The output of the cluster analysis was visualized with DISTRUCT (Rosenberg 2004).

The number of populations of *P. azorica* (*n*) which are necessary to represent 99.99 % of the total genetic diversity among populations (*P*) was calculated according to the modified equation of Ceska *et al.* (1997): *P* = 1−(*φ*_PT_)*^n^*.

## Results

### Genetic diversity

From 230 Azorean individuals distributed across 11 populations, the eight primers used yielded 79 clearly scorable bands, ranging from four at primer UBC836 to 15 at primers UBC807 and UBC842, with a mean value of 10. Among them 64 were polymorphic (81.0 %) (Table 2). At the island level, São Miguel presented the lowest percentage of polymorphic loci (54.4 %) and Pico presented the highest (63.3 %) (Table 3). At the population level, these values ranged from 17.72 % (CL from São Miguel) to 46.84 % (PB from Terceira). The total genetic diversity obtained by Shannon's information index (*I*) was 0.2456 and by computing Nei's genetic diversity (*h*) was 0.1503. While the population CL showed the lowest genetic diversity (*I* = 0.1131, *h* = 0.0795), the population PB showed the highest (*I* = 0.2421, *h* = 0.1619) (Table 3). All three indexes do not show significant differences (*P* = 0.455, *P* = 0.360, *P* = 0.321, respectively) within populations.

**Table 3.** Genetic diversity within 11 populations of *P. azorica. N*, sample size; *n*, number of polymorphic loci; *P%*, percentage of polymorphic loci; *N*_a_, number of different alleles; *N*_e_, number of effective alleles; *I*, Shannon's information index; *h*, Nei's gene diversity; SD, standard deviation.

Population	*N*	*n*	*P*%	*N*_a_ (SD)	*N*_e_ (SD)	*I* (SD)	*h* (SD)
S. Miguel							
Caloura	4	14	17.72	1.177 (0.384)	1.149 (0.333)	0.1131 (0.2476)	0.0795 (0.1750)
Lombo Gordo	16	28	35.44	1.354 (0.481)	1.242 (0.366)	0.2008 (0.2858)	0.1370 (0.1991)
Ribeira Quente	37	30	37.97	1.380 (0.488)	1.206 (0.338)	0.1810 (0.2660)	0.1203 (0.1844)
Tronqueira	7	23	29.11	1.291 (0.457)	1.161 (0.301)	0.1456 (0.2446)	0.0959 (0.1673)
Total	64	43	54.43	1.544 (0.501)	1.225 (0.328)	0.2173 (0.2525)	0.1380 (0.1759)
Pico							
Piedade	27	33	41.77	1.418 (0.496)	1.195 (0.315)	0.1822 (0.2549)	0.1183 (0.1749)
Santo Amaro	24	25	31.65	1.317 (0.468)	1.155 (0.273)	0.1493 (0.2402)	0.0970 (0.1613)
Alto São Roque	14	27	34.18	1.342 (0.477)	1.173 (0.295)	0.1629 (0.2484)	0.1062 (0.1687)
Cais do Mourato – Santana	37	36	45.57	1.456 (0.501)	1.215 (0.305)	0.2066 (0.2595)	0.1339 (0.1760)
Candelária	17	27	34.18	1.342 (0.477)	1.184 (0.296)	0.1720 (0.2548)	0.1130 (0.1723)
Total	119	50	63.29	1.633 (0.485)	1.190 (0.267)	0.2085 (0.2230)	0.1260 (0.1524)
Terceira							
Pico do Boi	19	37	46.84	1.468 (0.502)	1.275 (0.358)	0.2421 (0.2848)	0.1619 (0.1973)
Serreta	28	33	41.77	1.418 (0.496)	1.242 (0.355)	0.2113 (0.2775)	0.1409 (0.1918)
Total	47	47	59.49	1.595 (0.494)	1.269 (0.340)	0.2558 (0.2618)	0.1645 (0.1826)
Total (all islands)	230	64	81.01	1.810 (0.395)	1.233 (0.300)	0.2465 (0.2307)	0.1503 (0.1640)

### Population genetic structure

**Figure 1.**
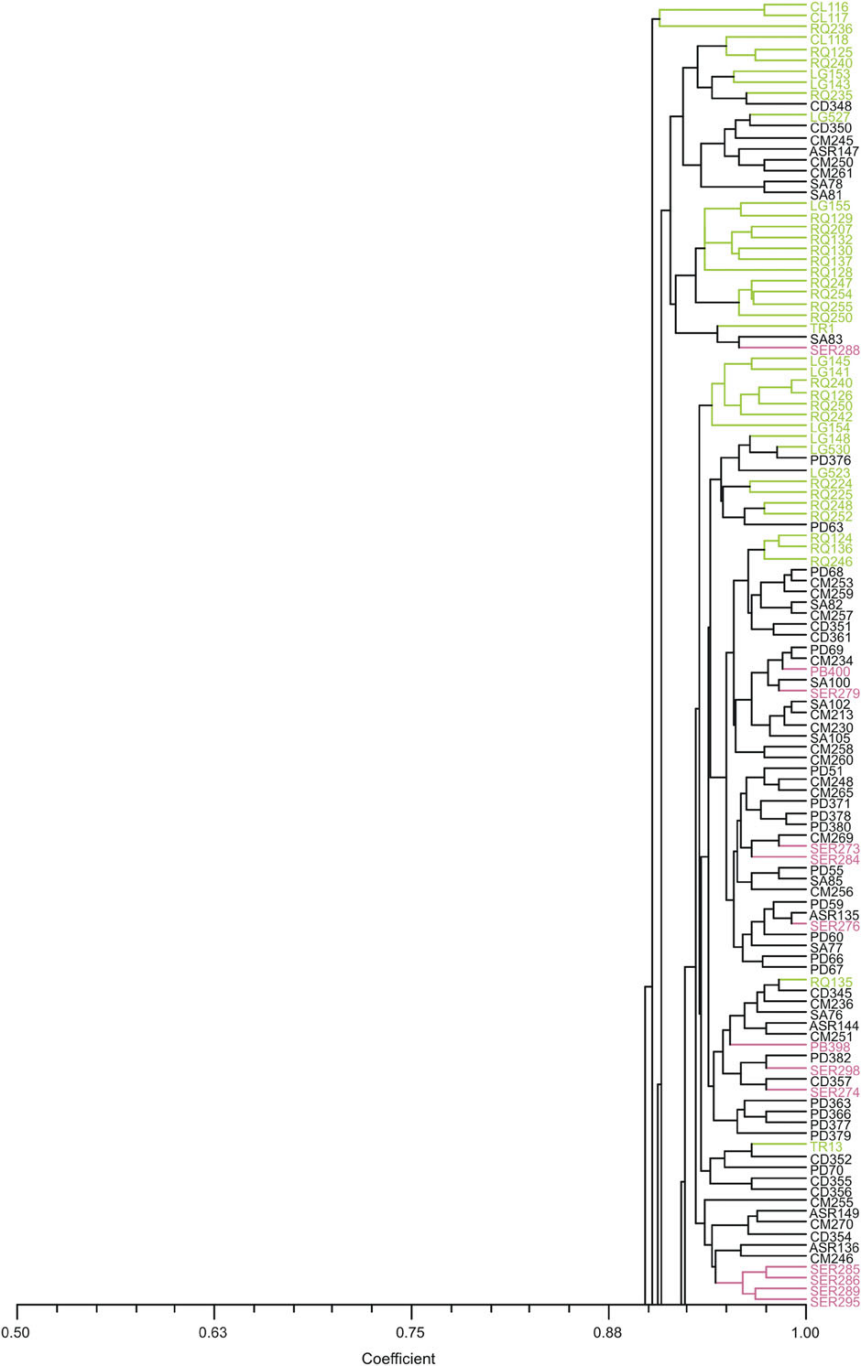

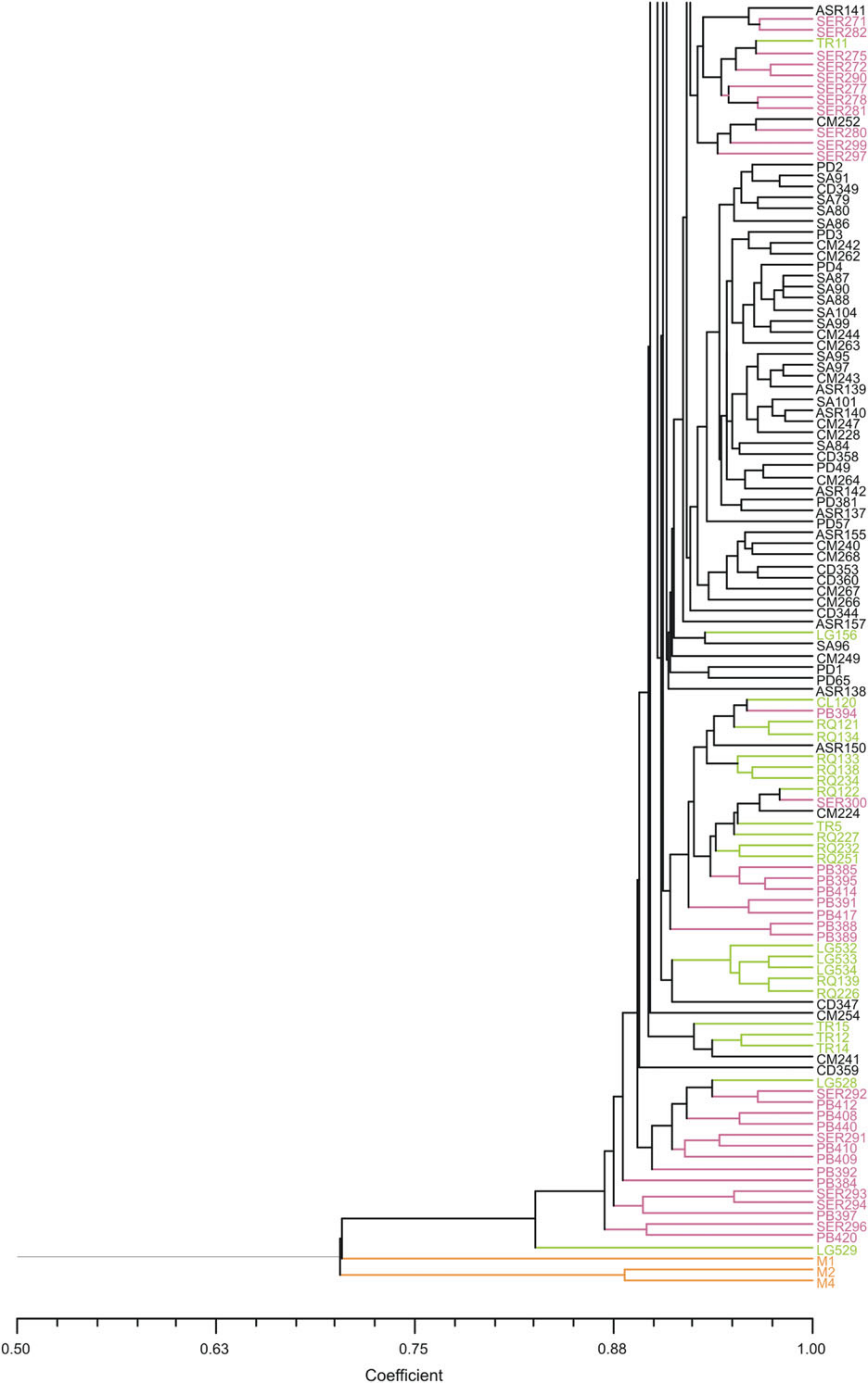
Relationship of 230 *P. azorica* genotypes from three islands in the Azores: São Miguel (green), Pico (black) and Terceira (pink); and three *P. excelsa* samples from Madeira island (orange). UPGMA tree based on Dice's coefficient.

The phenogram of *Picconia* spp. (Fig. 1) based on Dice's coefficient showed a high genetic similarity among all *P. azorica* genotypes with a mean value of 91.2 % and a clear differentiation of this species from *P. excelsa* from Madeira island with a similarity <76.2 %. In the tree a distribution of the samples with no obvious clustering according to the population and/or island of provenience was observed.

The AMOVA analysis that considered the three islands showed that 84 % of the total variation occurred within populations and only 8 and 8 % occurred among populations and among islands respectively (Table 4). Individually for each island, the AMOVA analysis indicated that most of the molecular variation in Terceira exists among individuals within populations (83 %), with lesser amounts among populations (17 %). Higher values of molecular variation among individuals within populations were obtained for São Miguel (89 %) and Pico (96 %).

**Table 4.** Analysis of molecular variance (AMOVA) for 11 populations of *P. azorica* distributed across three Azorean islands.

Source of variation	d.f.	Sum of squares	Variance components	% total variance
Among islands	2	113.316	0.535	8
Among populations	8	124.546	0.517	8
Within populations	219	1250.547	5.710	84

Pairwise unbiased Nei's genetic distance (Table 5) showed among populations within Pico values <0.013 and within Terceira populations a genetic distance of 0.034. For São Miguel population pairs CL-RQ, LG-RQ and RQ-TR showed values of genetic distance <0.033 while population pairs CL-LG, CL-TR and LG-TR showed values >0.051.

**Table 5.** Genetic differentiation and unbiased Nei's genetic distance among populations of *P. azorica*. Genetic differentiation (*φ*_PT_) is listed below the diagonal and unbiased Nei's genetic distance is listed above the diagonal.

Population	Caloura	Lombo Gordo	Ribeira Quente	Tronqueira	Piedade	Santo Amaro	Alto São Roque	Cais do Mourato	Candelária	Serreta	Terra Brava
Caloura	–	0.0514	0.0255	0.0563	0.0576	0.0838	0.0791	0.0679	0.0611	0.0592	0.0784
Lombo Gordo	0.184	–	0.0244	0.0634	0.0339	0.0472	0.0451	0.0366	0.0393	0.0399	0.0621
Ribeira Quente	0.082	0.080	–	0.0323	0.0256	0.0496	0.0427	0.0332	0.0348	0.0367	0.0487
Tronqueira	0.190	0.236	0.157	–	0.0271	0.033	0.0379	0.0269	0.0305	0.0297	0.0437
Piedade	0.281	0.119	0.158	0.155	–	0.0134	0.0126	0.0095	0.0086	0.0164	0.0535
Santo Amaro	0.339	0.177	0.226	0.169	0.068	–	0.0073	0.0109	0.0085	0.0246	0.0653
Alto São Roque	0.279	0.124	0.174	0.140	0.033	0.016	–	0.011	0.0109	0.0178	0.0571
Cais do Mourato	0.296	0.132	0.179	0.144	0.036	0.049	0.000	–	0.0097	0.0184	0.0512
Candelária	0.247	0.138	0.151	0.085	0.045	0.035	0.022	0.038	–	0.0179	0.0595
Serreta	0.280	0.176	0.207	0.162	0.105	0.165	0.066	0.112	0.091	–	0.0336
Terra Brava	0.194	0.221	0.192	0.117	0.201	0.282	0.178	0.212	0.189	0.138	–

Among all populations, genetic distance values <0.04 were detected for all population pairs, except for population pairs LG–SA, LG–ASR, RQ–SA and RQ–ASR (0.047, 0.045, 0.050, 0.043, respectively) and for populations CL from São Miguel and PB from Terceira. While population CL showed genetic distances >0.058 for all pairwise comparisons with populations from Pico and Terceira, population PB showed values >0.044 for all pairwise comparisons with populations from São Miguel and Pico.

**Figure 2.**
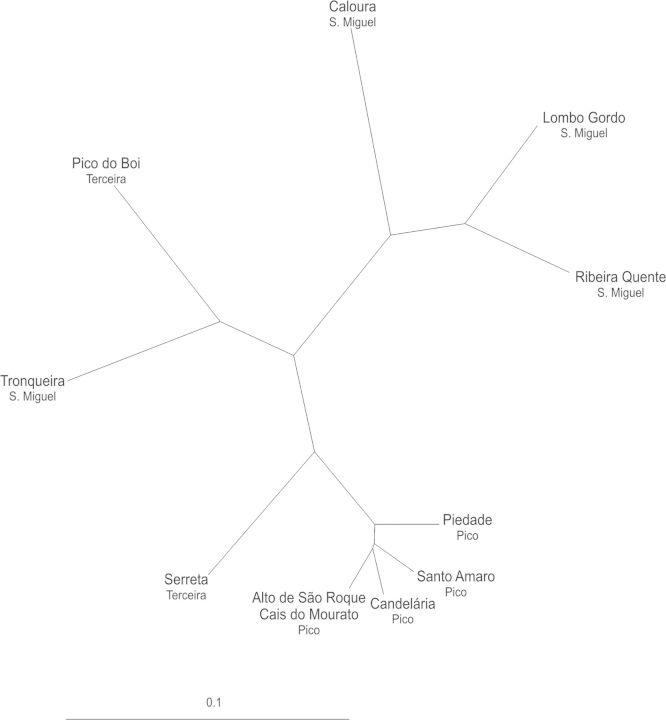
UPGMA radial tree showing the genetic relationships based on pairwise *φ*_PT_ values among 11 populations of *P. azorica*, occurring in three islands from the Azores.

The pairwise values of genetic differentiation (*φ*_PT_) (Table 5) between populations from São Miguel island varied between 0.080 for population pair RQ–LQ and 0.236 for population pair TR–LG; for Pico island ranged from 0.000 for population pair ASR–CM to 0.068 for population pair SA–PD; and for Terceira island it was 0.138 (PB–SER). Between islands the highest value (0.339) was observed between populations CL from São Miguel and SA from Pico. The determined values of *φ*_PT_ among islands showed levels of genetic differentiation of 0.118 (Pico–Terceira), 0.131 (São Miguel–Terceira) and 0.128 (Pico–São Miguel). The UPGMA dendrogram obtained with *φ*_PT_ pairwise values (Fig. 2) grouped the populations into four main clusters, one corresponding to populations RQ, LG and CL all from São Miguel island, another containing one population from Terceira (PB) and a population from São Miguel (TR), a third one containing population SER from Terceira and a fourth one with all populations from Pico (CM, ASR, CD, SA and PD).

**Figure 3.**
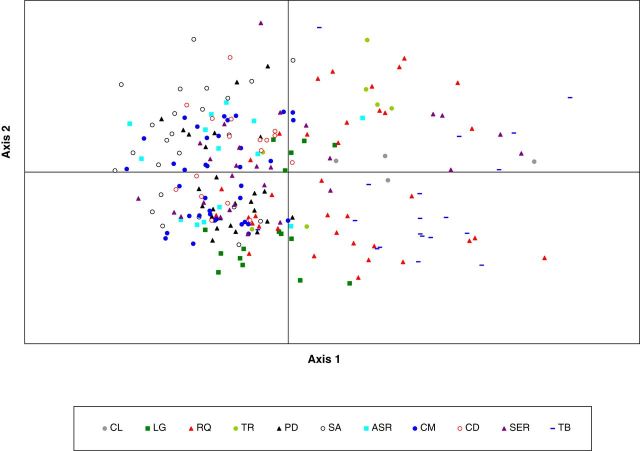
Two-dimensional representation of the first two axes of the principal component analysis from the matrix of genetic distances of 230 samples from 11 populations. Percentage of variance accumulates on the first two axes = 44.46 % (Axis 1 = 23.82 %; Axis 2 = 20.34 %). For population names see Table 1.

In the PCoA based on the matrix of individual genotypes, only 44.16 % of the total variance was explained by the first two axes (Axis 1 = 23.82 %; Axis 2 = 20.34 %) (Fig. 3) where most of the samples from Pico island cluster together on the left side of the plot reflected the genetic differentiation detected by *φ*_PT_.

The coefficient of genetic differentiation between populations (*G*_ST_) was 0.0940 and the level of gene flow (*N_m_*) calculated based on *G*_ST_ was estimated to be 4.818, suggesting a high gene flow between *P. azorica* populations and islands. The number of migrants calculated as described by Frankham *et al.* (2002) showed higher values among populations from Pico than among populations from the other two islands. The lowest value obtained among populations from Pico (3.438) was higher than the highest value obtained in the remaining islands (2.859). The estimated number of migrants between São Miguel and Terceira was 1.656, between São Miguel and Pico was 1.705 and between Pico and Terceira was 1.874.

Insignificant correlation between the genetic distance of individuals and geographical distances (*r^2^* = 0.047, *P* = 0.001) was shown by the Mantel test. For pairwise *φ*_PT_ and geographical distances a slightly positive correlation was obtained (*r^2^* = 0.242, *P* = 0.005).

The Bayesian analysis carried out in STRUCTURE for all populations demonstrated that there were no predominant genetic clusters. Average mean values of ln *P*(*K*) did not show any substantial increases when *K* varied from 1 to 14 **[see Supporting Information]**. Similar results were obtained when the analysis was performed separately for each island.

According to the genetic differentiation *φ*_PT_ value (0.17), the conservation of six populations is the minimum necessary in order to preserve 99.99 % of the total diversity of the 11 analysed populations.

## Discussion

Inter-simple sequence repeat markers have been used in population genetic studies of plant species as they effectively detect very low levels of genetic variation (Zietkiewicz *et al.* 1994). They are also potentially useful for analysing biogeographical patterns among populations within species (Bussell *et al.* 2004). With these purposes eight ISSR markers were successfully used to study the Azorean endemic plant species *P. azorica,* using 230 samples collected on populations across the Azorean islands of São Miguel, Terceira and Pico so as to provide knowledge of the levels and distribution of genetic diversity within these islands to help in designing conservation strategies for this endemic species.

The number of scored bands and the percentage of polymorphic loci obtained in this study can be considered enough for estimating genetic diversity within populations. Nybom (2004) who conducted an analysis on a compilation of different DNA markers for estimating intraspecific genetic diversity in plants calculated an average of 54.9 polymorphic markers per study. The values of genetic diversity for all populations obtained by Shannon's index (*I*) and Nei's gene diversity (*h*) were higher for the former but highly correlated (*r*^2^ = 0.010; *P* = 0.988). The estimated total diversity of *I* = 0.2465, *h* = 0.1503 was not expected as endemic and narrowly distributed plants usually show lower levels of genetic diversity and higher levels of genetic structure compared with their relatives with wider distribution areas (Hamrick and Godt 1989; Nybom 2004). The fact that the species is wind pollinated and zoochory (Arteaga *et al.* 2006; Dias *et al.* 2007) together with its long life cycle may explain the detected level of diversity. Long-lived species generally have a higher potential for long-range gene movement (Nybom and Bartish 2000). Similar results were obtained for this species when other populations were characterized with SSR molecular markers (Martins *et al.* 2013), which showed a total genetic diversity (*H*_T_) of 0.7. It is not surprising that Martins *et al.* (2013) obtained such a high level of total genetic diversity, as one of the SSR markers used detected a total of 31 alleles in 443 samples, indicating the presence of alleles at very low frequencies. Also other species from the *Oleae* complex, *Olea europaea* ssp. *cerasiformis* and *O. europaea* ssp. *Guanchica* living in insular habitats, in Madeira and the Canary Islands, respectively, also showed relatively high levels of total genetic diversity (García-Verdugo *et al.* 2009). Nevertheless, care should be taken when comparing data between studies as genetic diversity depends on numerous factors, namely life history, breeding system, growth life forms, geographical range and type of molecular method used (Powell *et al.* 1996; Nybom 2004). For example, the molecular markers used by Ferreira *et al.* (2011) are usually applied to identify genetic variances among different taxon and therefore limited intra-specific genetic diversity, and the absence of genetic structure was detected. Also Martins *et al.* (2013) analysed a wider geographical range by studying populations from eight islands and the number of samples per populations varied considerably. According to Nybom (2004), the number of plants per population has a positive effect on gene diversity values, probably because larger sample sizes may increase data quality.

The total genetic diversity was similar across islands, being slightly higher for Terceira, which also showed the higher genetic differentiation among populations due to the uneven distribution of alleles. Among all populations, the detected genetic diversity is evenly distributed with values higher for CM (*I* = 0.207, *h* = 0.134) from Pico, and lower for CL (*I* = 0.113, *h* = 0.080) and TR (*I* = 0.146, *h* = 0.096) from São Miguel. Population CM is one of the largest in terms of individuals and area of distribution; population CL is a very small isolated population highly disturbed by human activities; and TR is composed of a few dispersed individuals in a preserved natural forest of high altitude the conditions of which are considered to be out of the optimum range for the species (Schaefer 2003). When using the Pearson correlation analysis, we did find correlation (*P* = 0.05) between population size (log transformed) and genetic diversity when all populations were analysed, but when the smallest population (CL) was removed from the analysis correlations were not detected. Therefore, the diversity index from this population can be influenced by its reduced size (*N* = 4). The same analysis performed between islands and considering all populations did not find any correlation between population size and genetic diversity. Therefore, we have no indication that habitat fragmentation resulted in a pronounced loss of genetic diversity within *P. azorica* populations. If populations are small and isolated from one another with increased habitat fragmentation, the genetic flow could be capable of influencing the genetic structure and decreasing differentiation among populations (Ellstrand and Elam 1993). Our gene diversity data are strongly correlated (*P* = 0.023; *r*^2^ = 0.859) with the data obtained with SSR markers (Martins *et al.* 2013) for five of the common populations analysed. Population Santo Amaro (*n* = 24) from our study which corresponds to population Praínha (*n* = 2) from Martins *et al.* (2013) was not included in this analysis due to the discrepant number of samples analysed. The reasons for the highest diversity observed in PB population from Terceira, although occurring at high altitude, could be due to the difficulty in access and therefore less or no human disturbances. These factors have been described as the most suitable for successful long-distance dispersal in Macaronesia (Vargas 2007), and could have minimized human disturbances. Also the phenogram based on Dice's coefficient showed a high genetic similarity among all *P. azorica* genotypes with no obvious clustering according to population and/or island. The only clear differentiation is between *P. azorica* from the Azores archipelagos and *P. excelsa* from Madeira island.

For the endemic *P. azorica*, the results of AMOVA revealed that for each island most of the genetic diversity was found within populations (83 % for Terceira, 88 % for São Miguel and 96 % for Pico), a trend commonly reported in outcrossing and/or perennial species (Hamrick *et al.* 1992). Within and among islands, a low level of genetic differentiation was detected. Similar patterns of low differentiation among populations have also been reported in other insular trees of Macaronesia as *Morella faya* and *Morella rivas-martinezzi* (González-Pérez *et al.* 2009), where most of the genetic variability was found within populations (92 and 86 %, respectively). The genetic differentiation detected is a result of the high gene flow between populations and islands, corroborated by the number of migrants calculated, indicating that geographical distance was not found to be responsible for the reduction in gene flow between the different locations. Also levels of gene flow above one migrant between populations per generation were determined by Martins *et al.* (2013). Moreover, the lower value of total genetic diversity detected for Pico island reflects the continuous distribution of plants (Wright 1949) and a possible combination of founder and bottleneck effects, as Pico is the youngest island from Azores, and is caused by human interference that this species experienced.

The Mantel test failed to reveal isolation by distance, as no genetic structure was observed for the species. Also the Bayesian approach implemented in STRUCTURE corroborates these results, indicating that geographical distances that separate the central and eastern islands do not seem to have acted as barriers preventing gene flow (maximum interisland distance roughly 246 km). A possible explanation could be that several bird species, including the two Azorean endemic bird taxa *Columba palumbus azorica* and *Pyrrhula murina*, feed on its fruits (Dias *et al.* 2007) and as a result may have given an important contribution to *P. azorica* dissemination within and among islands, although the contribution of *P. murina* can be considered limited since it only exists in São Miguel. Regarding the results obtained here, it may also be considered that wind flow could provide further opportunities for long-distance dispersal of pollen.

Due to the over-exploitation of *P. azorica* and the habitat degradation that led to its extinction in Graciosa island, this species has been considered as a priority Azorean endemic species for conservation and measures for restoration of depleted natural populations should be taken. Moreover, *P. azorica* is part of the habitat of the second most threatened bird in Europe—the Azores bullfinch (*P. murina*) protected under the Birds directive (Directive 2009/147/EC of the European Parliament and of the Council of 30 November 2009 on the conservation of wild birds) and therefore knowledge about *P. azorica* can indirectly contribute to the preservation of other species. However in order to guarantee sustainable survival of populations and to preserve their evolutionary potential, knowledge of the levels of genetic diversity and their distribution is important for designing conservation strategies for threatened and endangered species (Hamrick and Godt 1989; Francisco-Ortega *et al.* 2000). Population establishment and long-term persistence as well as long-term evolutionary potential of restored populations is ensured by within-population genetic diversity (McKay *et al.* 2005), as loss of genetic diversity can lead to a decrease in the species' ability to survive environmental changes and demographical fluctuations both in short and in long term (Ellstrand and Elam 1993). Although the conservation of six populations is considered the minimum necessary in order to preserve 99.99 % of the total diversity of the analysed populations, management should aim to conserve as many of the small populations as possible. Concentrating conservation efforts only on the few large populations would result in the likelihood of loss of genetic variability for the species.

For long term, the most suitable strategy for the conservation of *P. azorica* is the protection and restoration of its habitat. Also artificial propagation of the species for timber use should be considered as the best preservation guaranteeing its *ex situ* conservation and sustainable survival, thus enhancing the *in situ* conservation. This could be sustainably achieved by propagation of seedlings and vegetative micropropagation of cuttings (D. Mendonâça *et al.*, submitted) and the reintroduction of the micropropagated plants into their populations of origin. Its valuable wood has been used in former times for the construction of toys, agricultural tools, furniture and religious statuary, and recent studies on its technological features support its use (Caetano-Ferreira *et al.* 2012). The establishment of a breeding programme for wood quality together with plant tissue culture techniques and micropropagation would for sure avoid the destruction of the small populations and the extinction of the species, fostering its use.

## Conclusions

Population genetic analysis with ISSR markers in the endangered endemic species *P. azorica* detected a high within-population genetic diversity but low genetic differentiation between populations and between islands which can be explained by its life history traits, dispersal and gene flow. Neither isolation of some habitats nor population size affected genetic variability within the studied populations. The obtained data are important for establishing guidelines and priorities for germplasm and genetic diversity conservation, with *in situ* and *ex situ* conservation, together with renewed economic interest for its utilization, following reforestation programmes, representing good opportunities for conservation of this tree species.

## Sources of Funding

This project was supported by the Azorean Government (Secretary for Natural Resources). LA IBB-CBA is supported by the Portuguese Foundation for Science and Technology (FCT, PEst-OE/EQB/LA0023/2013) and the Azorean Regional Science Fund (FRC). The authors thank A. Arraiol for providing samples of *Picconia excelsa* from Madeira Island. The following authors were supported by FRC: M.S.L. (M3.1.7/F/023/2011), D.M. (M3.1.7/F/010A/2009), S.X.B. (M3.1.7/F/026/2011) and A.R.B. (M3.1.2/F/039/2011), and by FCT: C.M. (SFRH/BPD/78059/2011).

## Contributions by the Authors

A.C.M. conceived the experiment. D.M., S.X.B. and C.M. collected the samples. M.S.L., A.R.B., C.M. and C.B. extracted the DNA. M.S.L. and A.R.B. performed the PCR and data analysis together with D.M. and S.X.B. M.S.L., D.M., S.X.B., A.R.B. and A.C.M. wrote the manuscript. All authors read the manuscript and agreed to submit it.

## Conflicts of Interest Statement

None declared.

## Supporting Information

The following Supporting Information is available in the online version of this article –

**Figure S1.** Location of the samples collected from São Miguel Island.

**Figure S2.** Location of the samples collected from Pico Island.

**Figure S3.** Location of the samples collected from Terceira Island.

**Figure S4.** Bayesian clustering performed by STRUCTURE for a set of 230 *P. azorica* genotypes from 11 different populations. (A) Δ*K* calculated as Δ*K* = *m*|*L*″(*K*)|/*s*[*L*(*K*)]; (B) ln Pr(*G|K*) values presented as a function of the number of clusters; (C) graphical presentations of different samples. Each sample is represented by a single vertical line broken into *K* colour segments, with lengths proportional to the estimated membership of the inferred cluster. Individuals are grouped into populations. For populations names see Table 1.

Additional Information
