# Supplementary material for: Genetic diversity of an Azorean endemic and endangered plant species inferred from inter-simple sequence repeat markers
Source: AoB Plants. 2014 Jun 26;6:plu034. doi: 10.1093/aobpla/plu034 (PMC4124485; doi:10.1093/aobpla/plu034)
Supplement: Additional Information [file supp_6_plu034_index.html]

Genetic diversity of an Azorean endemic and endangered plant species inferred from inter-simple sequence repeat markers — Additional Information 

# Genetic diversity of an Azorean endemic and endangered plant species inferred from inter-simple sequence repeat markers

## Additional Information

Additional Information

**Files in this Data Supplement:**

- Additional Information - pptx file
