# Supplementary material for: Genetic diversity of an Azorean endemic and endangered plant species inferred from inter-simple sequence repeat markers
Source: AoB Plants. 2014 Jun 26;6:plu034. doi: 10.1093/aobpla/plu034 (PMC4124485; doi:10.1093/aobpla/plu034)
Supplement: Additional Information [file supp_plu034_plu034supp.pptx]

## Slide 1
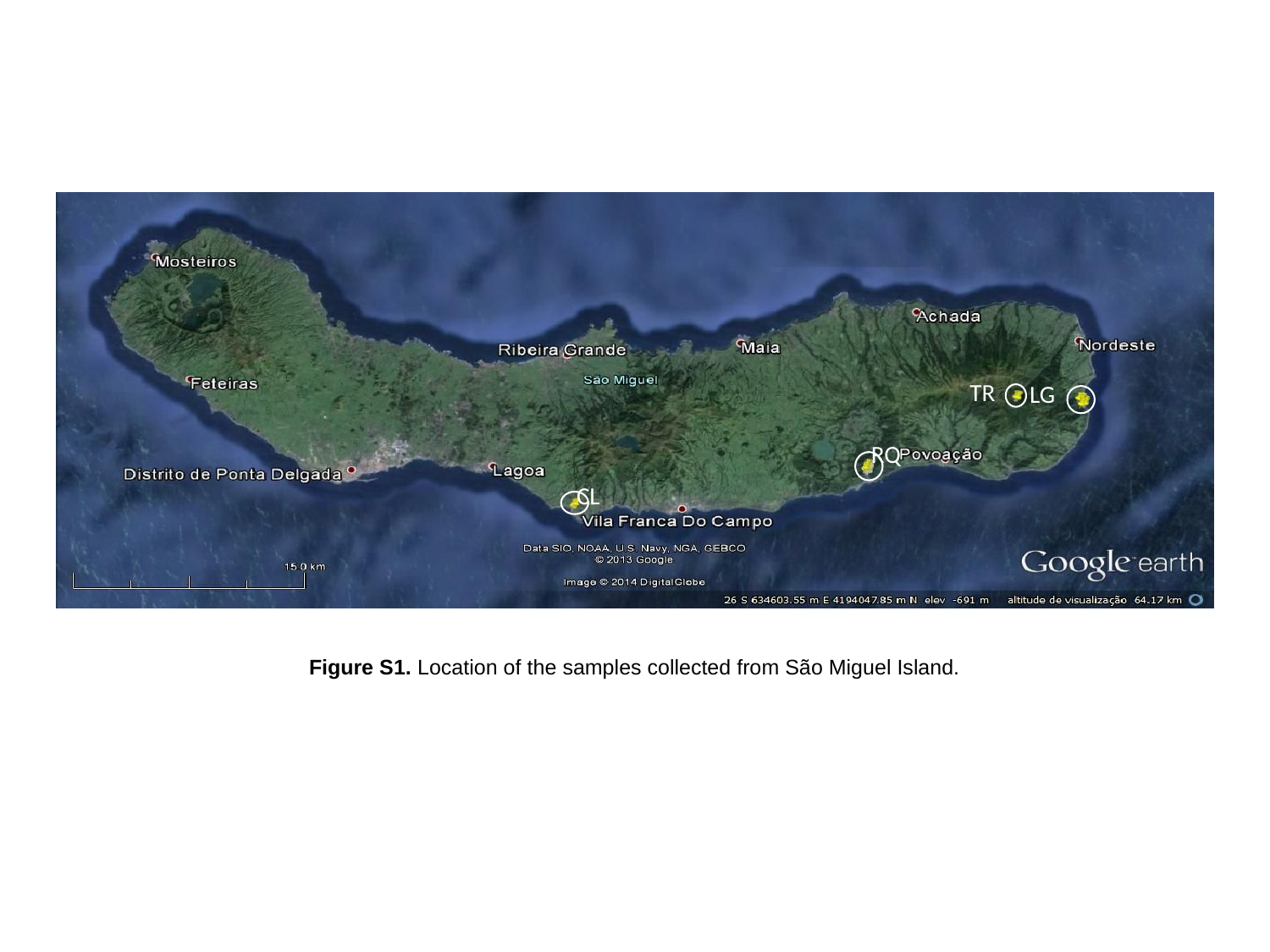

TR
LG
RQ
CL
Figure S1. Location of the samples collected from São Miguel Island.

## Slide 2
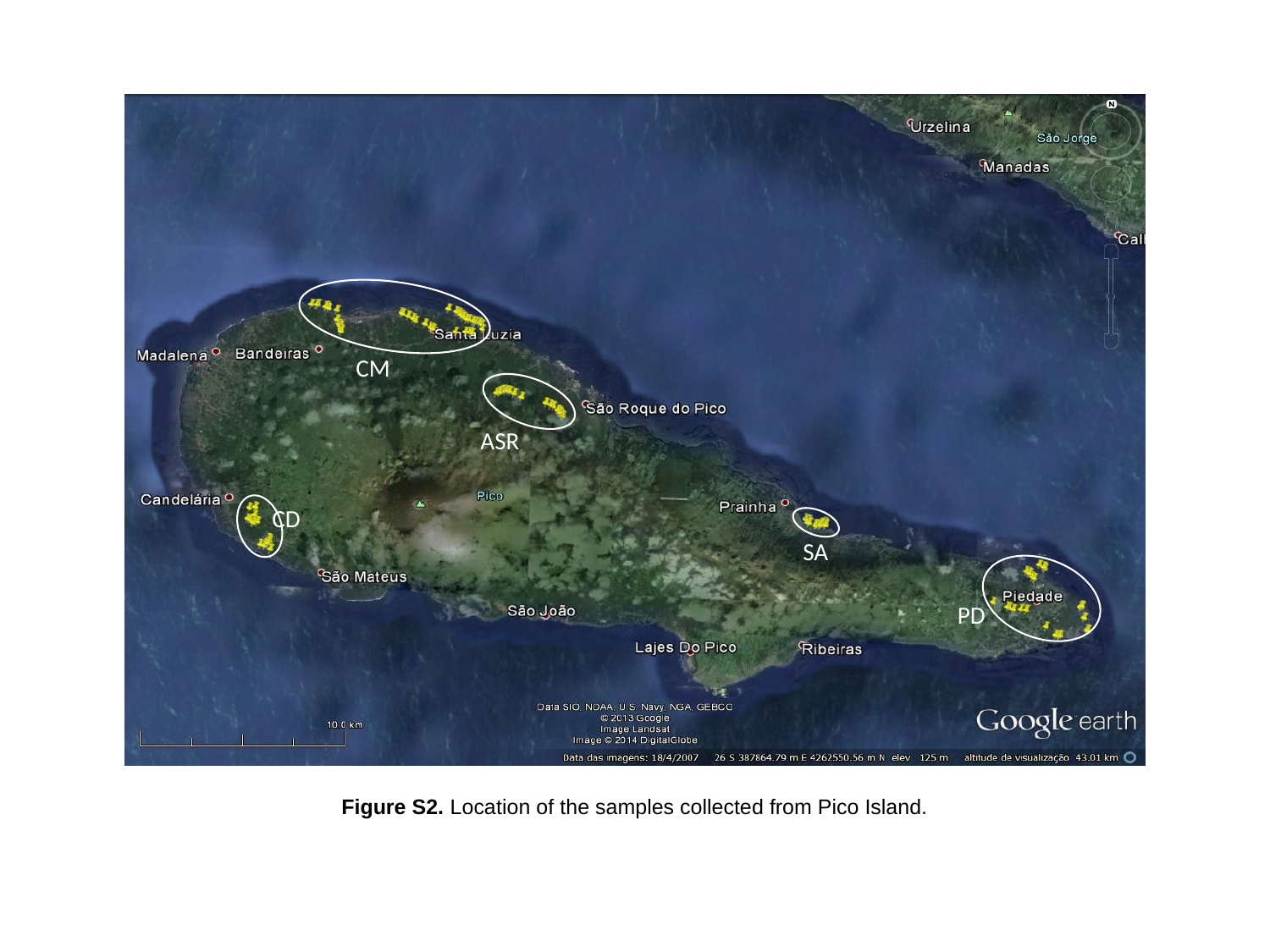

CM
ASR
CD
SA
PD
Figure S2. Location of the samples collected from Pico Island.

## Slide 3
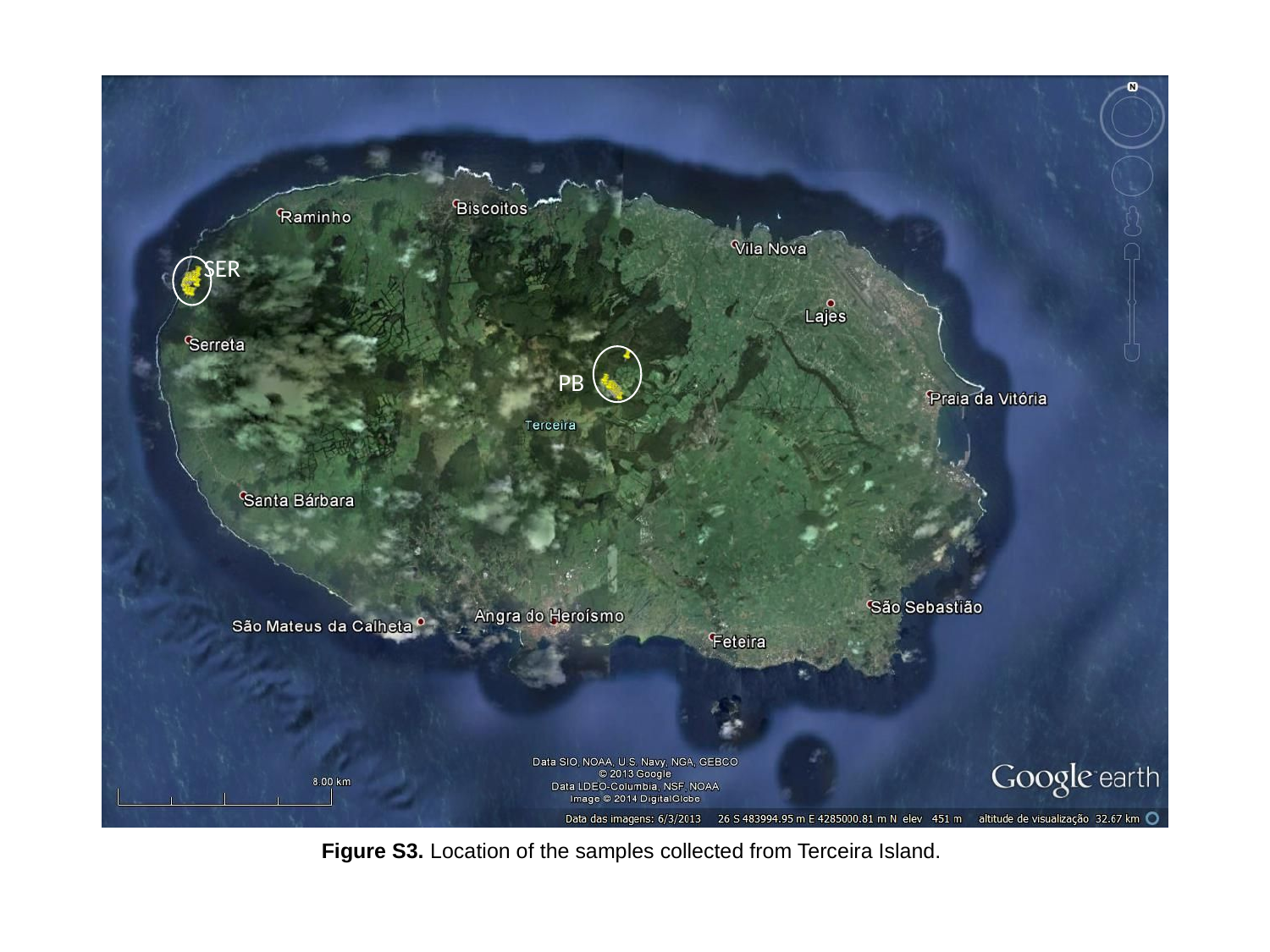

SER
PB
Figure S3. Location of the samples collected from Terceira Island.

## Slide 4
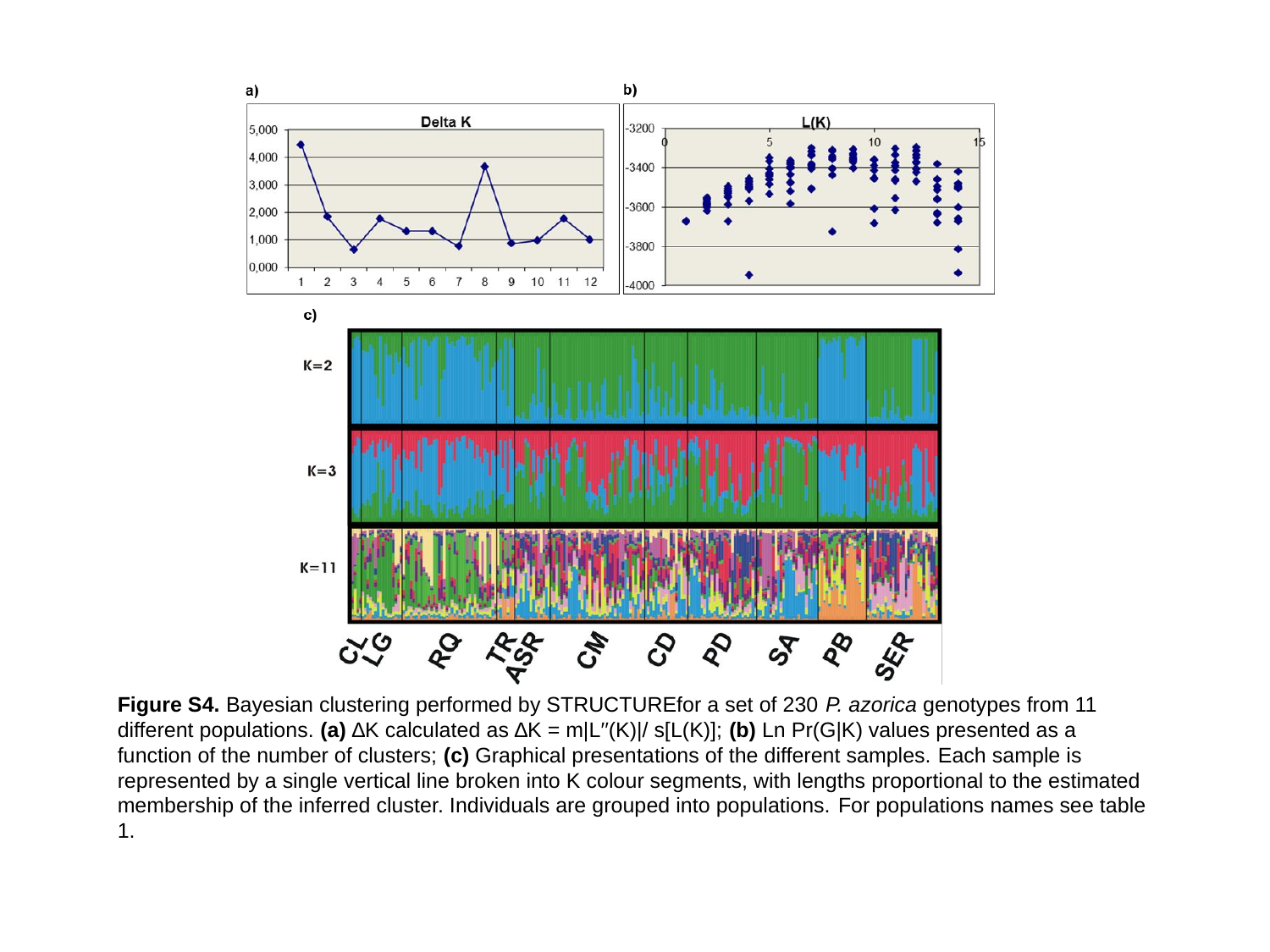

Figure S4. Bayesian clustering performed by STRUCTUREfor a set of 230 P. azorica genotypes from 11 different populations. (a) ∆K calculated as ∆K = m|L′′(K)|/ s[L(K)]; (b) Ln Pr(G|K) values presented as a function of the number of clusters; (c) Graphical presentations of the different samples. Each sample is represented by a single vertical line broken into K colour segments, with lengths proportional to the estimated membership of the inferred cluster. Individuals are grouped into populations. For populations names see table 1.
